# Severe hemoptysis: etiologies, management, and outcomes from a single-center experience over the last decade

**DOI:** 10.1186/s13613-025-01558-5

**Published:** 2025-09-25

**Authors:** Julien Dessajan, Aude Gibelin, Matthias Barral, Nina de Montmollin, Vincent Labbé, Michel Djibré, Guillaume Voiriot, Matthieu Turpin, Antoine Parrot, Muriel Fartoukh

**Affiliations:** 1 Service de Médecine Intensive Réanimation, Sorbonne Université, Assistance Publique-Hôpitaux de Paris (AP-HP), Hôpital Tenon, 4, rue de la Chine, Paris, 75020 France; 2 Service de Pneumologie et Oncologie Thoracique, Sorbonne Université, Assistance Publique-Hôpitaux de Paris (AP-HP), Hôpital Tenon, Paris, France; 3 Service de Radiologie, Sorbonne Université, Assistance Publique-Hôpitaux de Paris (AP-HP), Hôpital Tenon, Paris, France; 4 Sorbonne Université, Centre de Recherche Saint-Antoine UMR_S 938 INSERM, Paris, France; 5 Faculté de Médecine de Créteil, Université Paris Est Créteil, IMRB GRC CARMAS, Créteil, 94010 France

**Keywords:** Hemoptysis, Intensive care unit, Endovascular procedure, Pulmonary artery, Treatment outcome

## Abstract

**Background:**

Data on severe hemoptysis (SH) in the intensive care unit (ICU) remain scarce. We aimed to describe its clinical characteristics, etiologies, management strategies, and outcomes. This retrospective observational study analyzed patients admitted for SH to a referral center between 2009 and 2019. Data were compared to a historical cohort (1995–2009) using the Cochran–Armitage test.

**Results:**

A total of 945 patients (75% males; median age 55 years [IQR 42–65]) were analyzed; 67% had respiratory comorbidities. Invasive mechanical ventilation was required in 13% within 24 h of ICU admission. Lung cancer was the leading cause of SH, followed by bronchiectasis, tuberculosis, pneumonia, and aspergillosis. Compared with the historical cohort, pneumonia-related hemoptysis increased (11% vs. 5%; *P* < 0·001), as did pulmonary arterial involvement (12% vs. 5%; *P* < 0·001), mainly associated with pneumonia (23%), cancer, or aspergillosis (each 20%). Vascular interventional radiology (VIR) was first attempted in 81% of cases, achieving bleeding control in more than 90% of cases. Major adverse events occurred in 4.4% of cases. Emergent surgical lung resection (within 72 h) was performed in 2% of cases, all after VIR. In-hospital mortality rate increased slightly (8.7% vs. 6.5%; *P* = 0.08).

**Conclusions:**

Over the past decade, lung cancer became the leading cause of SH, with pneumonia increasingly contributing to pulmonary arterial involvement, reinforcing the need for multi-detector computed tomography angiography (MDCTA) screening. The high success rate of VIR confirms its key role, while surgery remains limited to rare cases. In-hospital mortality slightly increased, driven by a higher proportion of lung cancer.

**Supplementary Information:**

The online version contains supplementary material available at 10.1186/s13613-025-01558-5.

## Background

Severe hemoptysis (SH) is a life-threatening condition, with mortality rates reaching up to 50% without adequate treatment [1]. A recent French nationwide study found over 15,000 adults hospitalized annually for hemoptysis, with 10% requiring intensive care unit (ICU) admission and mortality rates of 9% at discharge and 27% at three years [2]. A study from our group identified factors associated with in-hospital mortality, such as chronic alcoholism, cancer, aspergillosis, pulmonary arterial involvement, and invasive mechanical ventilation [3]. A risk stratification score was developed, but has not yet been externally validated (see e-Table 1 in supplemental material) [3]. Over the past decade, interventional radiology—particularly bronchial artery embolization (BAE)—has played an increasingly important role in SH management and may have contributed to changes in therapeutic strategies, especially in referral centers [4]. Aside from a recent study focusing on BAE timing in Japan, there are few information on patients with SH admitted to the ICU [5]. The aims of this study were to describe changes in clinical characteristics, etiologies, management, and outcomes of adults with SH admitted to the ICU in a French referral center for SH, and to validate the risk stratification score [3].

## Methods

This observational study included all consecutive patients with SH admitted to the ICU of a tertiary university hospital and referral center for hemoptysis in Paris, France, from March 26, 2009, to March 29, 2019. Data were extracted from a prospective database and supplemented with medical charts if needed. Exclusion criteria were iatrogenic bleeding, bleeding of gastrointestinal or oropharyngeal origin and incomplete data. Alveolar hemorrhage and chronic hemoptysis (> 5 days) were also excluded, as their management may differ from that of acute hemoptysis. For recurrent hemoptysis episodes, only the first one was considered during the study period.

### Patient characteristics

For each patient, baseline characteristics were recorded. The initial severity of hemoptysis was assessed on ICU admission, as previously reported [6, 7] (see the online data supplement). The cause of hemoptysis was based on medical history, physical exams, chest radiographs, multi-detector computed tomography angiography (MDCTA), fiberoptic bronchoscopy when performed, and available microbiology and histology. Hemoptysis was considered cryptogenic when no cause was identified by the end of hospitalization or, if applicable, after outpatient follow-up [7]. The mechanism of hemoptysis was categorized as systemic (bronchial or non-bronchial systemic hypervascularization), pulmonary (pulmonary arterial involvement), or both. Pulmonary arterial involvement was diagnosed on MDCTA or pulmonary angiography [8].

### Management

Our approach favored conservative measures and vascular interventional radiology (VIR) over emergent surgery, whenever possible [9]. All therapeutic interventions—conservative measures, VIR, and thoracic surgery—were recorded during the hospital stay. Technical failures of endovascular procedures were defined as issues compromising procedure completion (inability to catheterize the vessel, instability of the catheter tip with risk of non-targeted embolization), or failed systemic embolization or pulmonary artery vaso-occlusion (PA VOC). All adverse events were recorded, as previously described [10]. Additional details are provided in the online data supplement.

### Outcomes

Bleeding control, defined as bleeding cessation or bleeding under 50 ml, was assessed at day 30 after ICU admission. Bleeding recurrence was a new episode of bleeding of at least 50 ml, arbitrarily considered significant, or requiring therapeutic intensification or a new therapeutic procedure at day 30, as previously described [10]. In-hospital length of stay and mortality were assessed at hospital discharge. Follow-up data were censored at 3 years for assessing the 1-month, 1-year and 3-year mortality rates post-admission.

### Data from the period 1995–2009

Adding data from May 1995 to March 2009 previously reported by our group [3], we analyzed selected variables related to etiology, management and outcomes over a 24-year period, divided into five-year periods (1995–1999, 2000–2004, 2005–2009, 2010–2014 and 2015–2019).

### Statistical analysis

Data are expressed as median (quartile) or numbers (percentage). Quantitative and nominal variables were compared using the unpaired *t*-test or the Mann-Whitney *U*-test, and the Chi-squared test or Fisher’s exact test, as appropriate. Comparisons with the historical cohort were performed, using Cochran-Armitage test.

### Ethical considerations

The study complied with French law, which does not require Institutional Review Board approval or patient consent for observational, noninterventional, retrospective analyses. The database met the requirements of the Commission Nationale de l’Informatique et des Libertés, CNIL (www.cnil.fr).

## Results

### Patients’ characteristics

**Fig. 1:**
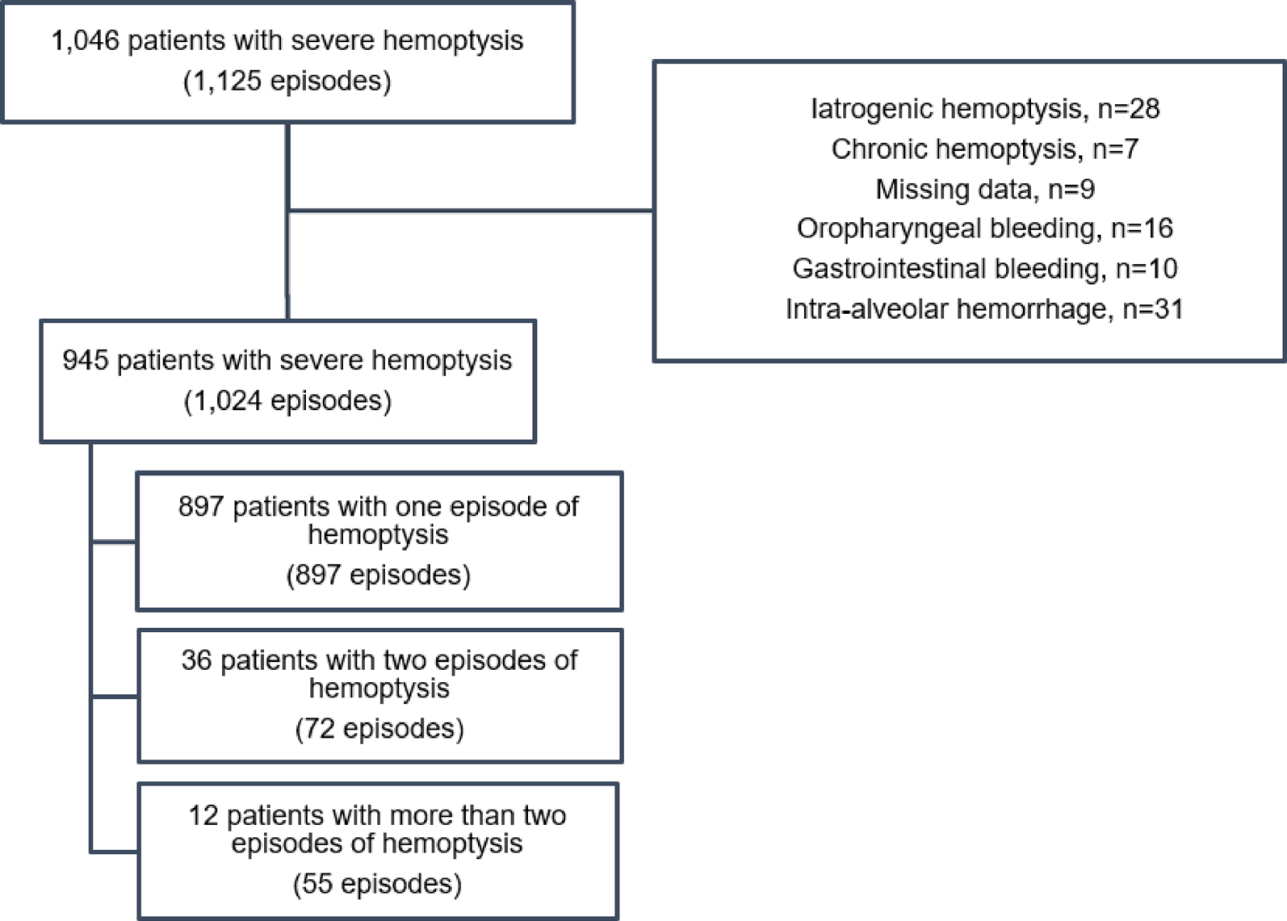
Selection of the population. The analysis focused on the first episodes of severe hemoptysis

Over the 10-year study period, 945 patients were admitted to the ICU for SH, 73% of whom being referred from other centers, mainly for VIR (Fig. 1). Patients had a median age of 55 years, and frequently presented comorbid conditions (*n* = 836; 89%), while keeping a good performance status (PS ≤ 1 in 85% of cases). Long-term therapy with anticoagulation or antiplatelet agent was administered to 258 patients (27%) (Table 1). Additional characteristics are available in e-Table 2.

**Fig. 2:**
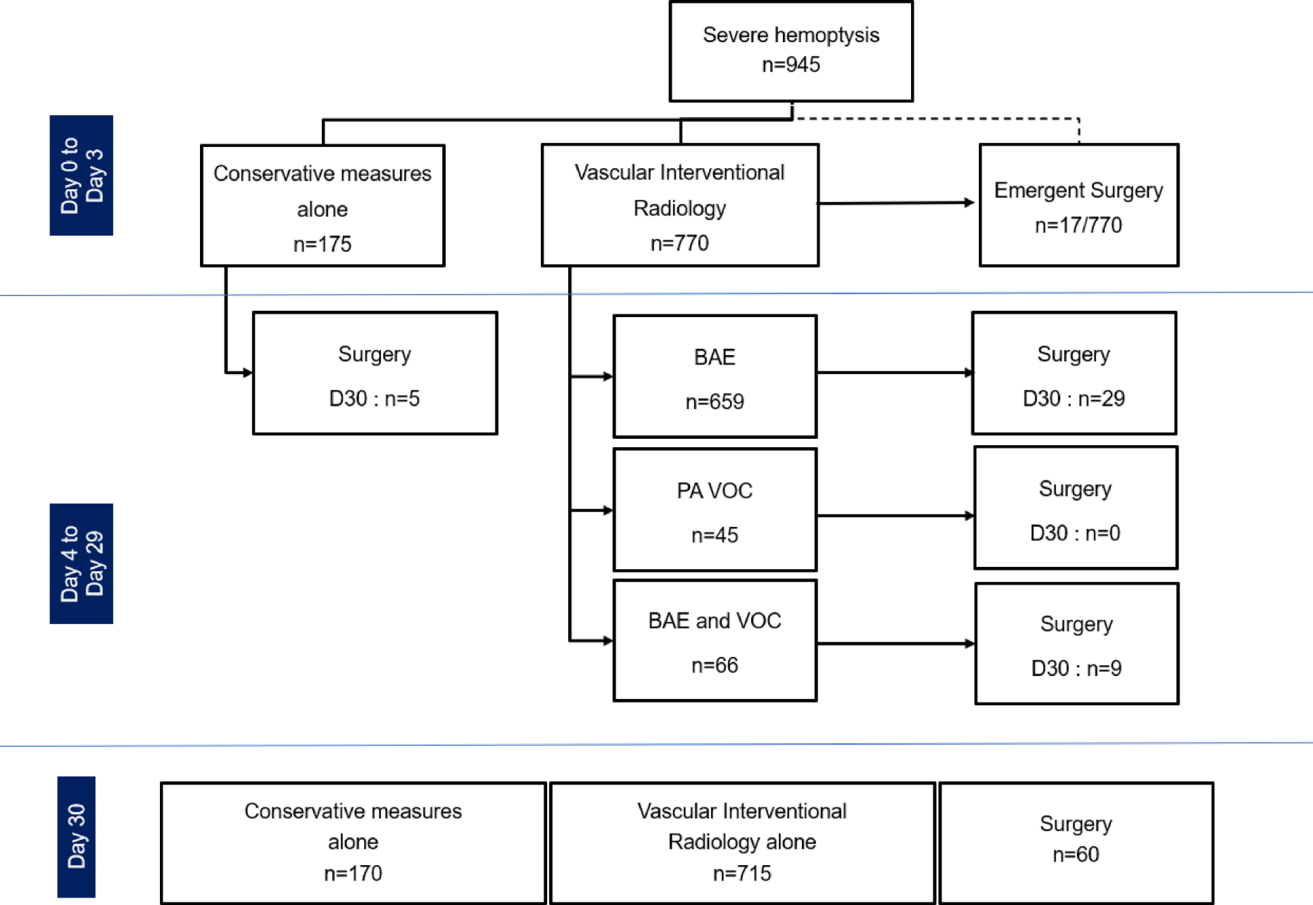
Management of severe hemoptysis.* BAE* bronchial arteriography with embolization,* PA VOC* pulmonary artery vaso-occlusion

**Fig. 3:**
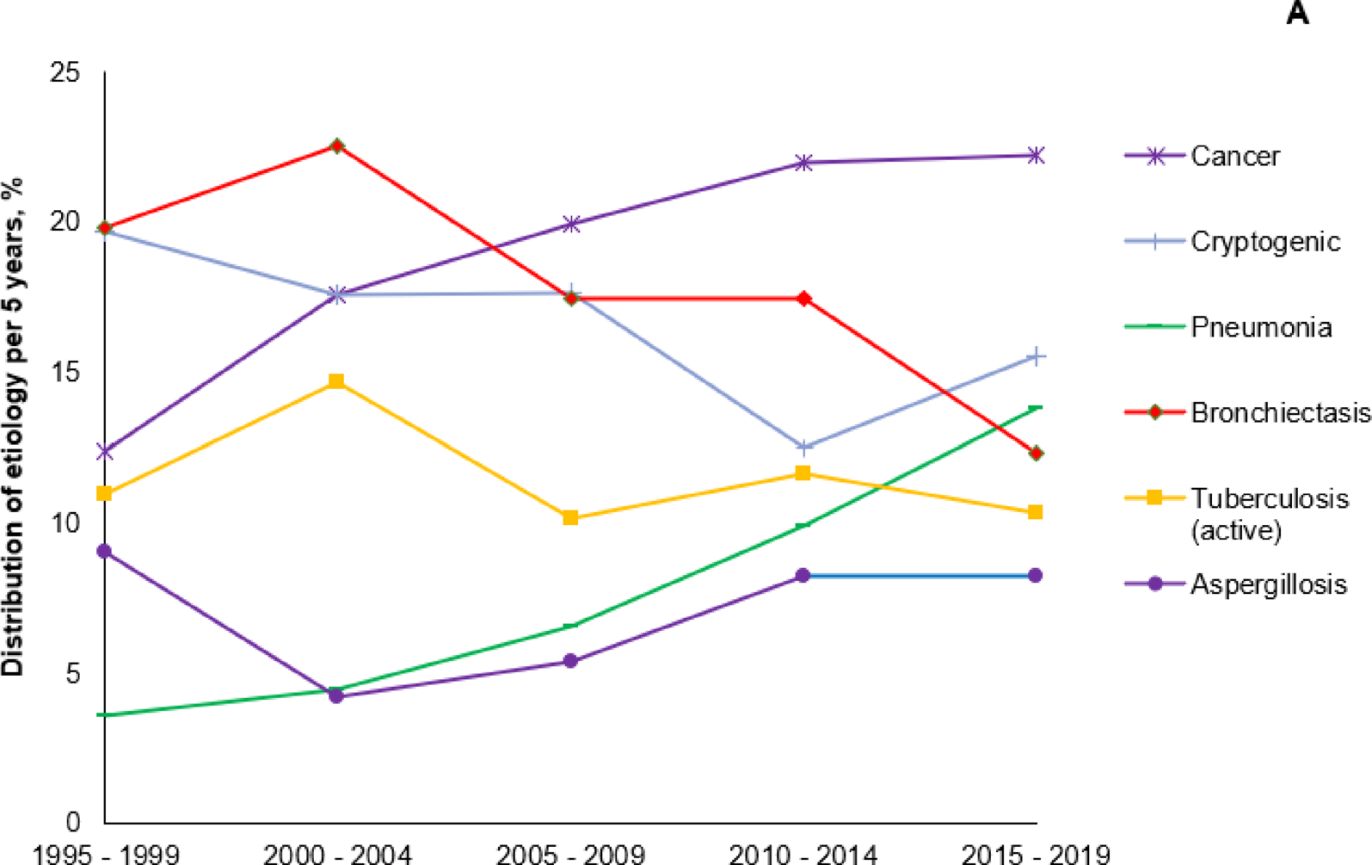

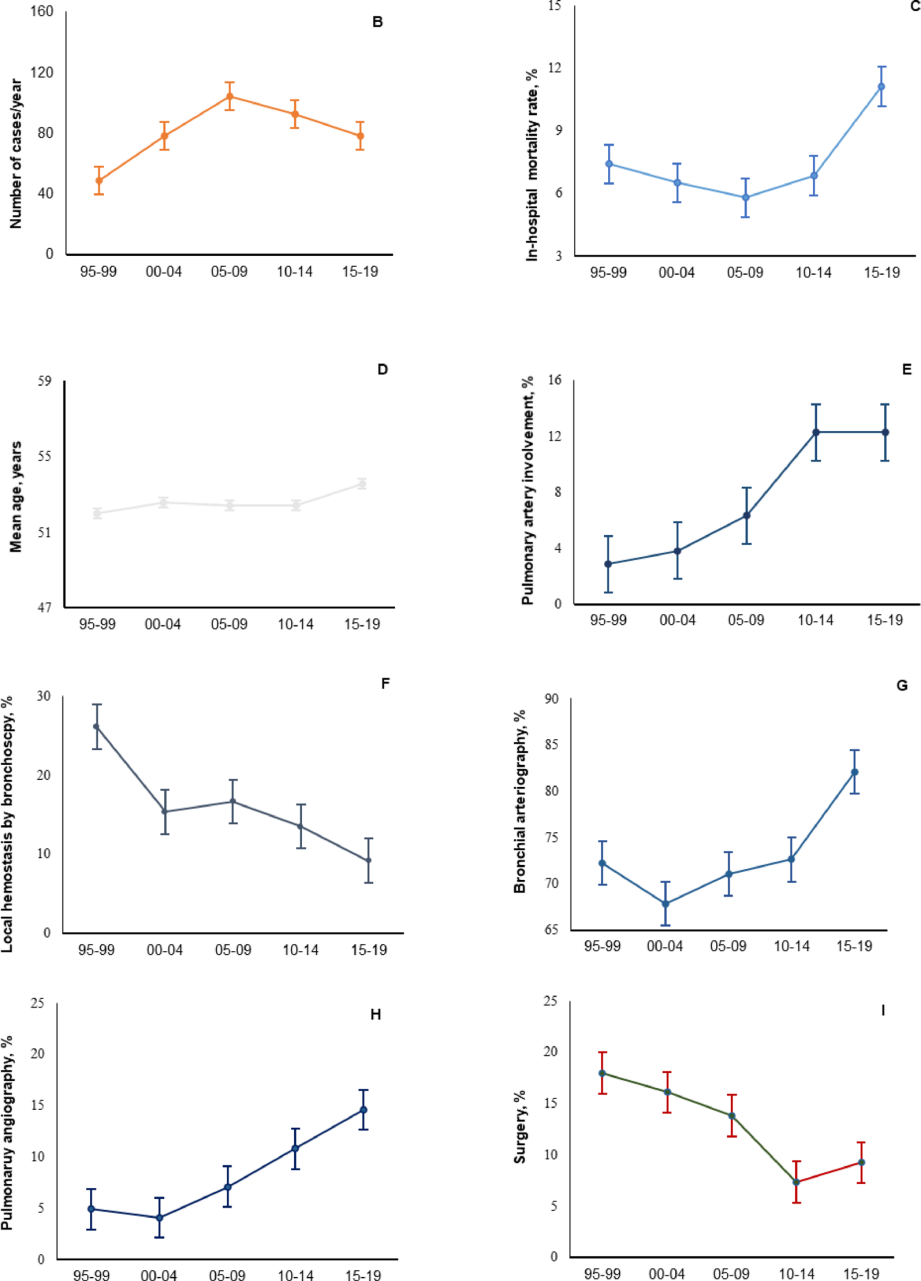
Data over a 24-year period (1995–2019). **A** Main etiologies of SH. **B** Number of cases per year. **C** In-hospital mortality rate. **D** Mean age. **E** Pulmonary arterial involvement. **F** Local hemostasis by bronchoscopy. **G** Bronchial arteriography; **H** Pulmonary angiography. **I** Surgery. Each point is the average of values over a 5-year period (1995: data available from May 12 to December 31. 2019: data available from January 1 to April 1), with error bars representing the standard error of the mean

### Hemoptysis characteristics

#### Initial severity of hemoptysis

The median expectorated blood volume was 170 ml [IQR 100–285] on ICU admission. Invasive mechanical ventilation was mainly indicated for acute respiratory failure before ICU referral (*n* = 81) and/or bleeding persistence or early recurrence (*n* = 39). Altogether, during the first 24 h of ICU admission, 15% of the patients required life support therapies, including invasive mechanical ventilation (*n* = 120; 13%), vasoactive drugs (*n* = 63; 7%) and cardiopulmonary resuscitation (*n* = 34; 4%); blood products transfusion was needed in 52 patients (6%) (Table 1). Initial biology is described in e-Table 3.

### Cause and mechanism of hemoptysis

Within 72 h of ICU admission, almost all patients were investigated with MDCTA (*n* = 939; 99%), whereas fiberoptic bronchoscopy (FOB) was performed in one half (*n* = 462; 49%) (e-Table 4). Lung cancer (*n* = 201; 21%) was the leading cause of SH, with 60 patients (30%) having no known cancer before the episode. Other frequent causes included bronchiectasis (*n* = 143; 15%), active tuberculosis (*n* = 108; 11%) and pneumonia (*n* = 104; 11%). The remaining causes were tuberculosis sequelae (*n* = 71; 8%), aspergillosis (*n* = 71; 8%), emphysema (*n* = 39; 4%) and pulmonary embolism (*n* = 11; 1%) (Table 2). No cause was identified in 145 episodes (15%). Overall, most episodes (*n* = 835; 88%) were related to bronchial and non-bronchial systemic hypervascularization alone. Pulmonary arterial vasculature was involved in 110 patients (12%), either alone or associated with systemic hypervascularization, particularly in those with pneumonia (23%), lung cancer (20%) and aspergillosis (20%) (Table 3). Both vasculatures were involved in 37 patients (4%). Etiologies and outcomes of patients with pulmonary arterial involvement are detailed in e-Table 5.

### Management

Conservative measures included discontinuation of drugs likely to worsen bleeding (*n* = 258; 27%), nebulization or local instillation of vasoconstrictor (*n* = 37; 4%) and/or administration of systemic terlipressin (*n* = 63; 7%). Inhaled tranexamic acid was not used during the study period. Bronchoscopic hemostatic tamponade was performed in 111 patients (12%). Antibiotics were administered to 590 patients (63%).

Within 72 h of ICU admission, 175 patients (18%) were managed using conservative measures only, and 770 patients (81%) were managed with VIR (Fig. 2). Of the latter, 17 (2%) patients had surgical lung resection performed in emergency (< 72 h of ICU referral) after successful VIR (*n* = 6), or clinical (bleeding recurrence, *n* = 7) or technical failure (*n* = 4) of VIR. In the former group, surgical lung resection alone was deemed indicated as first-line therapy in 5 patients (3 lung sequestration, 1 necrotizing pneumonia and 1 aspergilloma), after 8 days [7–9] days from ICU admission (respectively at 4, 7, 8, 9 and 13 days). Scheduled surgery was performed in 38 additional patients, during the ICU stay (*n* = 26), or planned after ICU discharge (*n* = 12). Altogether, at 30 days, surgery was required in 55 patients managed with VIR, after 6 days [3–12] from ICU admission, half of whom (*n* = 27) being operated in a context of clinical (*n* = 21) or technical failure (*n* = 6) of VIR. Altogether, at 30 days, additional sessions of VIR (2 sessions, *n* = 138; 3 sessions, *n* = 35; 4 sessions, *n* = 6; 5 sessions, *n* = 2) were attempted after a first attempt in 181 patients (24%) for non-controlled bleeding or bleeding recurrence, especially in those with aspergillosis and lung cancer. Vascular interventional radiology was associated with a bleeding control rate of 90% (*n* = 690), including BAE (*n* = 603/659), pulmonary angiography with vaso-occlusion (*n* = 37/45), or both (*n* = 50/66). Overall, clinical and technical failures of VIR were observed in 60 (6%) and 20 patients (2%), respectively. A summary of the therapeutic procedures performed during hospital stay is detailed in e-Table 6.

### Adverse events

Adverse events related to BAE occurred in 16% of cases (*n* = 115/725). Major adverse events included splenic or renal infarction, stroke, acute limb ischemia, myocardial infarction, and spinal infarction. No adverse event was related to pulmonary angiography and vaso-occlusion. Surgical lung resection performed in emergency (< 72 h of ICU referral, *n* = 17) or later within 30 days (*n* = 43) was associated with usual complications (*n* = 25/60; 42%) (e-Table 7).

### Outcomes

The need for initial invasive mechanical ventilation was particularly high in patients with lung cancer (22%) or pneumonia (28%), as compared with their counterparts (Table 3). Overall, 58 patients (6%) died in the ICU, one-third of whom (*n* = 20; 34%) within the first 72 h of ICU admission, and 86 patients (9%) died in the hospital (Table 4). Of note, ICU (*n* = 43/120; 36%) and in-hospital (*n* = 51/120; 42%) mortality rates were particularly high in patients mechanically ventilated within the first 24 h of ICU admission. Altogether, 34 patients (59%) died after withholding or withdrawing life sustaining therapies. In multivariable analysis, the factors associated with in-hospital mortality were a performance status > 2 (OR 5.92 [2.61–13.40], chronic alcoholism (OR 6.61 [2.13–20.48]), lung cancer (OR 4.80 [2.69–8.58], aspergillosis (OR 2.55 [1.04–6.26], CXR initial involvement (OR 3.23 [1.85–5.65], and invasive mechanical ventilation (OR 5.45 [2.94–10.10]. In-hospital mortality rates were close to those predicted by the score (e-Table 8). The ICU and in-hospital lengths of stay were 3 days [IQR 2–6] and 11 days [IQR 7–21], respectively. Follow-up data (censored at 3 years) were obtained for three quarters of patients (*n* = 716; 76%), with a mean and median follow-up duration of 755 ± 398 days and 990 days [IQR 379–1100], respectively. The 1-month, 1-year and 3-year mortality rates post-admission were 11.5% (92/802), 22.5% (158/703), and 36.5% (206/564), respectively (Fig. 3).

### Overall changes in characteristics, management and outcomes from 1995 to 2019 (Fig. 1)

While lung cancer was the third cause of SH (12%), behind bronchiectasis and cryptogenic hemoptysis (20% for both) during the period 1995–1999, the proportion of lung cancer markedly increased to 20% of all causes of SH during the past decade to become the leading cause of SH (Fig. 1A). Episodes of hemoptysis related to pneumonia also increased along time (1995–1999: 4%; 2015–2019: 14%; *P* < 0.001). Pulmonary arterial involvement was more frequently diagnosed along time, with a significant increase from 3% in 1995–1999 to 12% in 2010–2014 (*P* < 0.001) (Fig. 1E).

Altogether, the management of SH changed over time. Bronchoscopic tamponade procedures (1995–1999: 26%; 2015–2019: 10%; *P* = 0.006) were less often performed while endovascular procedures were increasingly used (BAE: from 72 to 82%; *P* < 0.001; and PA VOC from 5 to 14%; *P* < 0.001), and early surgical lung resection indications markedly decreased (from 18 to 9%; *P* < 0001) (Fig. 1F, G, H, I). The need for mechanical ventilation during the first 24 h remained similar along time: 11% between 1995 and 2009 and 13% between 2009 and 2019 (*P* = 0.23). The ICU mortality rate was stable between the two periods, while in-hospital mortality increased marginally (6.5% vs. 8.7%; *P* = 0.08) (Fig. 1C).

## Discussion

In this large retrospective cohort study, we provide an updated overview of the clinical presentation, etiologies, management, and outcomes of SH over the past decade. Compared with the historical cohort, MDCTA has supplanted FOB as the primary diagnostic tool, leading to an increased detection of pulmonary arterial vasculature involvement. Lung cancer became the leading cause of SH, while pneumonia-related cases rose substantially. Endovascular procedures were performed in most patients, with good results, while surgery was rarely performed in emergency settings.

Our cohort mainly included young men in good condition with respiratory and cardiac comorbidities, presenting with severe symptoms marked by large bleeding volumes and the need for mechanical ventilation. These findings align with a recent Canadian study [11].

Lung cancer has become the leading cause of SH, accounting for one in five patients, in line with trends from European and North American series [2, 11, 12]. In-hospital mortality among patients with cancer-related SH was 31% in a prior cohort from our team [13]. A recent study confirms a high rate of early bleeding recurrence and poor outcomes, especially with concurrent respiratory infections, frequently due to *Staphylococcus aureus* [14]. Therapeutic advances may have contributed to an increase in lung cancer patient admissions in our center [15]. In parallel, the rising incidence of lung cancer in France—particularly among women—may explain the increase in cancer-related SH. Women with cancer-related hemoptysis accounted for over 20% of cases in our cohort, up from 10% before 2000 [16]. Non-tuberculous pneumonia cases also rose, accounting for 14% of episodes compared to 4.3% in an earlier study from our team [17]. Bleeding from necrotizing forms was more challenging to control and often involved the pulmonary arterial vasculature. These trends mirror findings from an Italian study reporting pneumonia as the second most common cause of hemoptysis (19%) [12]. Cryptogenic hemoptysis accounted for 15% of cases, in line with recent series, reflecting progress in diagnostic techniques [2, 6, 18–21].

Pulmonary arterial involvement increased from 5 to 12% between the two decades, reflecting improved detection with MDCTA [3], which enables accurate diagnosis and better guides therapeutic strategies, including pulmonary vaso-occlusion [8, 22]. In pneumonia- and cancer-related episodes—both frequently involving the pulmonary arteries—MDCTA is particularly valuable [11, 23].

Although complementary, but MDCTA—due to its superior spatial resolution and diagnostic yield—should be prioritized for initial evaluation, reserving bronchoscopy for therapeutic intervention in active endobronchial bleeding [11]. In our cohort, half of the patients underwent urgent FOB, compared to 94% in earlier years [6]. Some studies suggest that performing bronchoscopy during the acute phase of hemoptysis, in addition to CT, does not improve the diagnostic yield for lung cancer [24]. Our findings support the role of MDCTA as the preferred initial diagnostic and pretherapeutic tool in the management of SH [25].

Regarding medical management, terlipressin use declined from 34 to 10%, reflecting improved collaboration with interventional radiologists, a shift toward endovascular-first strategies, and the limited evidence supporting its nebulized form at the time [6]. Inhaled tranexamic acid, a more recent addition to the therapeutic arsenal, was not used during the study period, as its broader adoption followed the publication of supporting evidence in 2018 [26].

Endovascular radiology played a central role, with 81% of patients receiving it as first-line treatment—comparable to other series of severe cases and markedly higher than in non-severe hemoptysis [2, 11, 12]. Technical failure rates declined from 20 to 8%, reflecting improved operator expertise and advances in super-selective catheterization and embolization techniques [6]. The overall success rate of BAE reached 91.5%, in line with a large review [27]. Recurrence rates remained variable, particularly in cancer- or aspergillosis-related cases, which are more prone to relapse [28–30]. Our 6% rate of major adverse events was comparable to previous reports (6.6%) [28].

Surgery was exceptionally indicated in emergency, and always following failed embolization. This likely reflects a shift in practice over the past decade, with increasing adherence to prior data suggesting that attempting VIR as first-line treatment may reduce the risk of postoperative complications [9].

Our in-hospital mortality rate (8.7%) was close to the 13.9% reported in a Canadian series of moderate-to-severe hemoptysis [11]. Between 1995 and 2009, Fartoukh et al. reported a 6.5% overall mortality and 32.5% among mechanically ventilated patients, as compared with 8.7% and 42% in our current series [3]. This slight increase may reflect a higher proportion of cancer-related episodes. Mortality rates were otherwise similar when excluding cancer patients (4.8% vs. 4.0%). Our overall in-hospital mortality remained low compared to previous SH series in high-income countries, possibly because nearly 75% of patients received early interventional radiology [11]. Early BAE has recently been associated with lower mortality in mechanically ventilated patients with hemoptysis [5].

The mortality observed in our cohort was consistent with predictions from the score developed by Fartoukh et al. [3].

Long-term outcomes remain less explored; in our cohort, one- and three-year mortality were 15% and 21%, with 9% recurrence over a 990-day median follow-up—comparable to French national data [2].

The main limitation of our study is its single-center design, which may have selected the most severe cases, as 73% of patients were referred from other centers after failed initial management. Detailed information on the type of referring service or initial care was not available in our dataset. However, our study represents one of the largest ICU-based SH cohort to date, spanning 24 years. Comprehensive data collection and reanalysis of imaging provided detailed insights into the etiology and mechanisms of hemoptysis, particularly the underreported pulmonary arterial involvement.

## Conclusions

In conclusion, this study provides an updated picture of patients with SH admitted to a referral center in France. Lung cancer has become the leading cause, and should be routinely screened. Clinicians should also assess for pulmonary arterial mechanism, which is increasingly common and potentially fatal if undiagnosed and untreated. Early identification of prognostic factors is another essential step in patients orientation and management. Thanks to its enhanced performance and high benefit-risk ratio, vascular interventional radiology plays a key role in the treatment of these patients, with surgery limited to rare to exceptional cases. Further multicenter studies clarifying the indication, timing and benefit-risk ratio of these procedures are needed to better guide clinicians.

**Table 1:** Main initial characteristics

Variables	Missing, *n*	
*Baseline characteristics*		
Age (years), median [IQR]	0	55 [42–65]
Male, n (%)	0	713 (75)
Performance status ≤ 1, n (%)	1	801 (85)
Tobacco use, n (%)	5	664 (70)
Chronic alcoholism, n (%)	87	271 (29)
Respiratory comorbid condition, n (%)	0	633 (67)
Cardiovascular comorbid condition, n (%)	0	344
Charlson score, median [IQR]	0	1 [0–3]
Charlson score > 1, n (%)		423 (45)
Long-term anticoagulation and/or antiplatelet agent, n (%)	0	258 (27)
History of hemoptysis, n (%)	0	224 (24)
*Hemoptysis characteristics*		
Cumulated expectorated volume (ml), median [IQR]	59	170 [100–285]
Hemoglobin (g/dl), median [IQR]	3	12.2 [10.5–13.9]
CXR quadrants, *n* ≥ 2	6	326 (34)
CT lobes, *n* ≥ 3	14	295 (31)
*Initial severity (first 24 h)*		
SAPS II, median [IQR]	0	19 [13–27]
Invasive mechanical ventilation, n (%)*	0	120 (13)
Vasopressors, n (%)	0	63 (7)
Blood products transfusion, n (%)	0	52 (6)
CPR, n (%)	0	34 (4)

Results are expressed as median [IQR] or number (%), unless otherwise stated

*CXR* Chest X-ray,* CT* Computed Tomography,* SAPS II* Simplified Acute Physiology Score, *CPR* cardiopulmonary resuscitation

*Initial invasive mechanical ventilation was mainly indicated for acute respiratory failure before ICU referral (*n* = 81) and/or bleeding persistence or early recurrence (*n* = 39). Two patients were intubated due to agitation to facilitate VIR procedures under general anesthesia, and one patient was ventilated specifically for surgery. All three were extubated within 24 h

**Table 2:** Etiology and mechanism of severe hemoptysis

Etiology	*n* (%)
Cancer*	201 (21)
Primitive pulmonary	178/201 (89)
Pulmonary metastasis	23/201 (11)
Bronchiectasis	143 (15)
Pneumonia	104 (11)
Active tuberculosis	108 (11)
Tuberculosis sequelae	71 (8)
Pulmonary aspergillosis	71 (8)
COPD, emphysema	39 (4)
Pulmonary embolism	11 (1)
Other causes	60 (4)
Cryptogenic	145 (15)
*Mechanism*	*n (%)*
Systemic bronchial and non-bronchial artery only	835 (88)
Pulmonary artery only	73 (8)
Both	37 (4)

Results are expressed as number (%)

COPD, chronic obstructive pulmonary disease

Other causes included pulmonary artery malformation (*n* = 8), sarcoidosis (*n* = 6) and other interstitial lung diseases (*n* = 5), and pulmonary hypertension (*n* = 2)

*Among the 201 patients with lung cancer-related hemoptysis, 60 (30%) had no known cancer prior to the episode of severe hemoptysis

**Table 3:** Characteristics and outcomes according to etiology

	All episodes (*n* = 945)	Cancer (*n* = 201)	Bronchiectasis (*n* = 143)	Cryptogenic (*n* = 145)	Tuberculosis, active (*n* = 108)	Tuberculosis sequelae (*n* = 71)	Pneumonia (*n* = 104)	Aspergillosis (*n* = 71)
*Characteristic*								
Age, years	53 ± 17	59 ± 12	58 ± 18	50 ± 15	37 ± 16	56 ± 16	56 ± 15	51 ± 16
Sex: male	713 (75)	159 (79)	82 (57)	110 (76)	89 (82)	54 (76)	83 (80)	56 (79)
History of respiratory comorbid condition	633 (67)	166 (83)	112 (78)	38 (26)	45 (42)	64 (90)	63 (61)	69 (97)
Hemoglobin, g/dl	12·0 ± 2·5	11·1 ± 2·3	12·5 ± 1·9	13·9 ± 1·8	12·0 ± 1·9	12·2 ± 2·5	10·9 ± 2·4	11·5 ± 2·8
Initial mechanical ventilation^‡ †^	120 (13)	45 (22)	4 (3)	6 (4·1)	7 (7)	7 (10)	29 (28)	7 (10)
Cumulated volume expectorated on ICU admission, ml	196 ± 186	195 ± 236	202 ± 130	188 ± 126	217 ± 176	250 ± 185	140 ± 147	208 ± 249
*Management*								
Bronchial arteriography	725 (77)	164 (82)	123 (86)	114 (79)	84 (78)	56 (79)	55 (53)	61 (86)
Pulmonary angiography	111 (12)	39 (19)	1 (0·7)	1 (0·7)	12 (11)	3 (4)	28 (27)	15 (21)
*Mechanism*								
Bronchial and non-bronchial systemic hypervascularization alone	835 (88)	160 (80)	142 (99)	145 (100)	94 (87)	68 (96)	80 (77)	57 (80)
Pulmonary artery involvement (alone or associated with systemic hypervascularization)	110 (12)	41 (20)	1 (0·7)	0	14 (13)	3 (4)	24 (23)	14 (20)
*Outcome*								
ICU mortality	58 (6·1)	33 (16·4)	1 (0·7)	2 (1·4)	1 (1)	4 (5)	9 (9)	6 (8)
In-hospital mortality	86 (9·1)	47 (23·4)	2 (1·4)	2 (1·4)	1 (0·9)	5 (7)	13 (12·5)	10 (14·1)
ICU length of stay, days	5 ± 11	5 ± 6	4 ± 3	6 ± 25	5 ± 3	5 ± 11	8 ± 8	6 ± 5
Hospital length of stay, days	19 ± 28	18 ± 22	11 ± 9	12 ± 40	27 ± 27	15 ± 28	27 ± 40	26 ± 26

Results are expressed as mean ± standard deviation or number of cases (%), unless otherwise stated

Missing data: Volume on ICU admission, cryptogenic (*n* = 2), cancer (*n* = 34), bronchiectasis (*n* = 5), tuberculosis (active) (*n* = 5), tuberculosis (sequelae) (*n* = 3), pneumonia (*n* = 17)

^†^ within 24 h of ICU admission

**Table 4:** Outcomes of 945 patients with SH

Overall population	Missing, *n*	
ICU mortality, n (%)	0	58 (6.1)
In-hospital mortality, n (%)	0	86 (9.1)
ICU length of stay (days), median [IQR]	0	3 [2–6]
In-hospital length of stay (days), median [IQR]	0	11 [7–21]
Bleeding control, n (%)		
At ICU discharge	0	878 (93)
At hospital discharge	2	872 (92)
Follow-up data available, n (%)	143	716 (76)
Duration of follow-up (days), median [IQR]*	143	990 [379–1100]
Recurrence at last follow-up, n (%)	447	91/498 (18)
1-month estimated mortality, n (%)	143	92/802 (11.5)
3-month estimated mortality, n (%)	174	111/771 (14.4)
1-year estimated mortality, n (%)	242	158/703 (22.5)
3-year estimated mortality, n (%)	381	206/564 (36.5)

Results are expressed as median [IQR] or number of cases (%)

*Censored at three years

## Supplementary Information

Below is the link to the electronic supplementary material.

Supplementary Material 1.

## Data Availability

The datasets generated and/or analyzed during the current study are not publicly available due to privacy and ethical restrictions but are available from the corresponding author on reasonable request. De-identified participant data, data dictionary, study protocol, and statistical analysis plan are securely stored at Sorbonne Université, Service de Médecine Intensive Réanimation, AP-HP Hôpital Tenon. Data will be accessible from the date of publication with no end date, subject to approval of a research proposal and signature of a data access agreement ensuring appropriate use.

## Abbreviations

BAE: Bronchial arteriography with embolization
CNIL: Commission Nationale de l’Informatique et des Libertés
ICU: Intensive care unit
MDCTA: Multi-detector computed tomography angiography
PA VOC: Pulmonary artery vaso-occlusion
PS: Performance status
SH: Severe hemoptysis
VIR: Vascular interventional radiology
