# Supplementary material for: Severe hemoptysis: etiologies, management, and outcomes from a single-center experience over the last decade
Source: Ann Intensive Care. 2025 Sep 25;15:137. doi: 10.1186/s13613-025-01558-5 (PMC12463777; doi:10.1186/s13613-025-01558-5)
Supplement: Supplementary file 1 — Supplementary Material 1. [file 13613_2025_1558_MOESM1_ESM.docx]

**ADDITIONAL FILE**

**Severe hemoptysis: etiologies, management, and outcomes from a single-center experience over the last decade**

Table of contents

[Study Design and Methods 2](#_Toc203490836)

[e-Table 1. Mortality risk score in severe hemoptysis 3](#_Toc203490837)

[e-Table 2. Detailed baseline characteristics and comorbidities of 945 patients with severe hemoptysis 4](#_Toc203490838)

[e-Table 3. Clinical and laboratory data on ICU admission 5](#_Toc203490839)

[e-Table 4. Diagnostic workup within the first 72 hours of ICU admission and results 6](#_Toc203490840)

[e-Table 5. Etiologies and outcomes of 110 patients with pulmonary arterial involvement 7](#_Toc203490841)

[e-Table 6. Therapeutic procedures performed during hospital stay 8](#_Toc203490842)

[e-Table 7. Adverse events 9](#_Toc203490843)

[e-Table 8. In-hospital mortality by severity score 10](#_Toc203490844)

# Study Design and Methods

The criteria for defining severe hemoptysis (SH) were the following: (i) bleeding volume >200 mL in less than 48 hours; (ii) acute respiratory failure: need for invasive mechanical ventilation, high-flow nasal oxygen (HFNO) therapy, or supplemental oxygen >6 L/min; (iii) pulmonary arterial involvement; (iv) mild-to-moderate hemoptysis (100–200 mL) uncontrolled despite medical treatment or VIR; and (v) uncontrolled hemoptysis despite medical treatment, with recurrence >50 mL within 6 hours.

***Management***

All patients received conservative measures. As the administration of systemic terlipressin may interfere with the success of BAE, its use was avoided whenever possible. Fiberoptic bronchoscopy was performed at referral when bleeding location was unclear on MDCTA or to administer topical treatment, using cold saline solution lavage or instillation of vasoconstrictive agents. Endovascular treatment was the first-line therapy for patients with severity criteria on admission. Whenever possible, MDCTA was performed before the endovascular procedure. In case of an unstable catheter or dangerous collaterals, super selective catheterism was performed using a microcatheter, as recommended. A pulmonary angiography with vaso-occlusion was performed in patients with hemoptysis with suspected pulmonary arterial involvement. It was associated with BAE in cases of persistent or recurrent bleeding after a first-line BAE. Emergent surgical resection was indicated in case of uncontrolled bleeding despite endovascular procedures. When performed after bleeding control, surgery was either scheduled during the same hospitalization, or secondarily planned (after hospital discharge).

*Local organization for the management of SH*

Given our ICU’s role as a regional referral center for SH, one bed was systematically reserved at the beginning of each night shift whenever possible. Night shifts are covered by both a senior intensivist and a resident, physically present in the unit, ensuring immediate evaluation and management of SH patients.

ICU admission was guided by the following criteria:

- Acute respiratory failure (need for invasive mechanical ventilation, HFNO, or oxygen ≥6 L/min),
- Large hemoptysis volume (>200 mL within 48 hours),
- Suspected pulmonary arterial mechanism (e.g., necrotic mass, excavated lesion, cancer, aspergillosis, Behçet disease),
- Active mild-to-moderate hemoptysis with recurrence >50 mL within 6 hours despite medical treatment,
- Mild-to-moderate hemoptysis in patients with significant comorbidities or specific therapeutic plans.

Patients with mild hemoptysis not meeting these criteria were initially managed in medical wards, with escalation to the ICU as needed.

*Vascular interventional radiology (IR): indications and timing*

Urgent VIR procedures (i.e., within first hours of ICU admission) were performed in case of:

- Acute respiratory failure,
- Large-volume bleeding (≥200 mL in <48 h),
- Recurrence >50 mL in <6 h despite medical management,
- Suspected pulmonary arterial mechanism.

Non-immediate VIR procedures (within 24 h) were considered for:

- Moderate hemoptysis (100–200 mL),
- Recurrent mild hemoptysis despite medical treatment.

Elective procedures were discussed in cases of:

- Mild and chronic hemoptysis,
- Preoperative planning.

# e-Table 1. Mortality risk score in severe hemoptysis

| **Variables** |  | **Points** |
| --- | --- | --- |
| Comorbid condition and habitus | Chronic alcoholism | 1 |
| Cause of hemoptysis | Lung cancer | 2 |
|  | Aspergillosis | 2 |
| Mechanism of hemoptysis | Pulmonary artery involvement | 1 |
| Abundance of hemoptysis | CXR quadrants ≥ 2 on admission | 1 |
|  | Initial invasive mechanical ventilation | 2 |

Abbreviations: CXR, chest X-ray.

From Fartoukh *et al.* (5)

# e-Table 2. Detailed baseline characteristics and comorbidities of 945 patients with severe hemoptysis

| **Variables** | **Missing, n** |  |
| --- | --- | --- |
| **Performance status (PS)** | 1 |  |
| PS 0 |  | 467 (49) |
| PS 1 |  | 334 (35) |
| PS 2 |  | 100 (11) |
| PS 3 |  | 35 (4) |
| PS 4 |  | 8 (0.8) |
| **Mac Cabe score** | 0 |  |
| Mac Cabe 1 |  | 712 (75) |
| Mac Cabe 2 |  | 133 (14) |
| Mac Cabe 3 |  | 100 (11) |
| **No comorbidity** |  | 109 (12) |
| **Tobacco use** | 5 | 664 (70) |
| active | 12 | 425 (50) |
| pack-years | 50 | 30 [20-45] |
| **Chronic alcoholism** | 87 | 271 (29) |
| **Respiratory history** |  | 633 (67) |
| Lung cancer |  | 165 (18) |
| primary pulmonary |  | 141 (15) |
| metastasis |  | 24 (3) |
| Tuberculosis |  | 195 (21) |
| sequelae |  | 68 (7) |
| COPD |  | 155 (16) |
| Bronchiectasis |  | 94 (10) |
| cystic fibrosis |  | 9 (1.0) |
| Pneumonia |  | 72 (8) |
| Aspergillosis |  | 60 (6) |
| Pulmonary embolism |  | 40 (4) |
| Other |  | 179 (19) |
| **Cardiac history** |  | 343 (36) |
| Ischemic heart disease |  | 105 (11) |
| Rhythmic heart disease |  | 78 (8) |
| Valvular disease |  | 26 (3) |
| High blood pressure |  | 242 (26) |
| **Peripheral arterial disease** |  | 76 (8) |
| **Stroke** |  | 38 (4) |
| **Diabetes** |  | 127 (13.4) |
| with complications |  | 52 (6) |
| **Dyslipidaemia** |  | 121 (13) |
| **Chronic renal failure** |  | 41 (4) |
| creatinine >200 μmol/l |  | 20 (2) |
| **Liver disease** |  | 67 (7) |
| Cirrhosis |  | 25 (3) |
| **Cancer** |  | 235 (25) |
| with metastasis |  | 109 (12) |
| **Leukaemia** |  | 7 (0.7) |
| **Lymphoma** |  | 17 (2) |
| **HIV infection** |  | 22 (2) |
| **Charlson score** | 0 | 1 [0-3] |
| **Promoting treatment** | 0 | 258 (27) |
| Anticoagulant |  | 107 (11) |
| Oral |  | 78 (8) |
| Heparin |  | 29 (3) |
| Antiaggregant |  | 167 (18) |
| Others (Bevacizumab) |  | 1 (0.2) |
| **History of hemoptysis** | 0 | 224 (24) |
| within the previous year |  | 142 (15) |

Results are expressed as median (IQR) or number of cases (%).

Abbreviations: CPOD, chronic obstructive pulmonary disease.

e-Table 3. Clinical and laboratory data on ICU admission

| **Variable** | **Missing, n** |  |
| --- | --- | --- |
| **Cumulated Volume on admission (ml)**, median [IQR] | 59 | 170 [100-285] |
| <50 ml |  | 57 (6) |
| [50 - 100] ml |  | 139 (15) |
| [100 - 200] ml |  | 262 (28) |
| >200 ml |  | 428 (45) |
| **SAPS II**, median [IQR] | 0 | 19 [13-27] |
| **Clinical features** |  |  |
| SBP, mmHg | 19 | 137 ± 27 |
| <100 mmHg, n (%) |  | 37 (4) |
| Heart rate, /min | 18 | 89 ± 20 |
| >130/min, n (%) |  | 25 (3) |
| Temperature, °C | 48 | 37 ± 0.7 |
| >38.5°C, n (%) |  | 40 (4) |
| **Respiratory support** | 0 |  |
| Room air |  | 436 (46) |
| Supplemental oxygen therapy |  | 429 (45) |
| NIV |  | 2 (0.2) |
| IMV |  | 78 (8) |
| **Blood gas results** |  |  |
| pH | 362 | 7.40 ± 0.7 |
| PaO2, mmHg | 359 | 102 ± 61 |
| PaCO2, mmHg | 359 | 41 ± 10 |
| HCO3-, mmol/l | 381 | 25 ± 4 |
| PaO2/FiO2 | 359 | 327 ± 116 |
| **Other laboratory parameters** |  |  |
| White blood cells, G/L | 5 | 11 ± 13 |
| Platelets, G/L | 7 | 259 ± 112 |
| <100 G/L, n (%) |  | 42 (4) |
| Haemoglobin, g/dl | 3 | 12.1 ± 2.3 |
| ≤ 9 g/dl, n (%) |  | 106 (11) |
| Prothrombin time, % | 75 | 86 ± 20 |
| ≤ 50%, n (%) |  | 44 (5) |
| aPTT ratio | 74 | 1.09 ± 0.24 |
| ≥ 1.5, n (%) |  | 45 (5) |
| Urea, mmol/L | 4 | 6 ± 4 |
| ≥ 10 mmol/L, n (%) |  | 79 (8) |
| Creatinine, μmol/L | 4 | 79 ± 60 |
| >120 μmol/L, n (%) |  | 66 (7) |

Results are expressed as mean ± standard deviation or median [interquartile range], or number of cases (%).

Abbreviations: SAPS II, Simplified Acute Physiology Score; SBP, systolic blood pressure; NIV, non-invasive ventilation; IMV, invasive mechanical ventilation; aPTT, activated partial thromboplastin time.

# e-Table 4. Diagnostic workup within the first 72 hours of ICU admission and results

| **Variable** | **Missing, n** | **n (%)** |
| --- | --- | --- |
| **Chest CT scan, n (%)** | 1 | **944 (99.9)** |
| Timing |  |  |
| Within the first 72 hours |  | 939 (99) |
| Post bronchoscopy |  | 46 (5) |
| Post interventional radiology |  | 23 (2) |
| Results |  |  |
| Lobes affected, n ≥ 3 | 14 | 295 (31) |
| Bleeding location | 14 | 806 (85) |
| Etiological orientation | 14 | 767 (81) |
| BSH | 44 | 661 (70) |
| NBSH | 44 | 110 (12) |
| PA involvement | 43 | 87 (9) |
| **Chest X-ray, n (%)** | **6** | **842 (89)** |
| ≥ 2 Quadrants involved |  | 326 (34) |
| **Fibreoptic Bronchoscopy, n (%)** |  | **490 (52)** |
| Timing |  |  |
| Within the first 72 hours |  | 462 (49) |
| After ICU discharge |  | 7 (1.2) |
| Results |  |  |
| Blood traces |  | 375 (77) |
| Active bleeding |  | 216 (44) |
| Clot |  | 187 (38) |
| Flooding |  | 93 (19) |
| Bud |  | 74 (15) |
| Local tamponade* |  | 111 (23) |
| Frozen serum |  | 78 (16) |
| Adrenalized lidocaine |  | 71 (15) |
| Local terlipressin |  | 45 (9) |
| **Microbiological samplings, n (%)** | | |
| Respiratory tract sample | 8 | 732 (78) |
| Sputum |  | 504 (53) |
| Tracheal aspirate |  | 198 (21) |
| PTC |  | 10 (2) |
| BAL |  | 15 (2) |
| Bacterial species | 8 | 337 (46) |
| *M. tuberculosis* |  | 75 (10) |
| *P. aeruginosa* |  | 72 (10) |
| *H. influenzae* |  | 40 (6) |
| MSSA/MRSA^§§^ |  | 39 (5) - 3 (0.4) |
| *Klebsiella* |  | 33 (5) |
| *E. coli* |  | 23 (3) |
| *Enterobacter* |  | 22 (3) |
| *S. pneumoniae* |  | 19 (3) |
| Other *Streptococcus* |  | 14 (2) |
| Other bacteria |  | 72 (10) |
| Testing for Aspergillus | 8 | 132 (14) |
| Negative tests |  | 85 (9) |
| Positive sputum culture |  | 32 (3) |
| Positive serology |  | 11 (1) |
| Positive antigenemia |  | 4 (0.5) |

Abbreviations: CT, Computed Tomography; BSH, bronchial systemic hypervascularization; NBSH, non-bronchial systemic hypervascularization; PA, pulmonary artery; PTC, plugged telescoping catheter; BAL, bronchoalveolar lavage; MSSA/MSRA, methicillin-susceptible/methicillin-resistant *Staphylococcus aureus*.

*Bronchoscopic hemostatic tamponade included endobronchial instillation of cold saline, adrenaline, and/or terlipressin.

# e-Table 5. Etiologies and outcomes of 110 patients with pulmonary arterial involvement

| **Variable** | **Missing, n** | **n (%)** |
| --- | --- | --- |
| **Etiology** | 0 |  |
| Cancer |  | 41 (37) |
| Pneumonia |  | 24 (22) |
| Pulmonary aspergillosis |  | 14 (13) |
| Active tuberculosis |  | 14 (13) |
| Tuberculosis sequelae |  | 3 (3) |
| Bronchiectasis |  | 1 (1) |
| Other causes* |  | 13 (12) |
| Behçet’s disease |  | 4 (4) |
| Hereditary haemorrhagic telangiectasia (Rendu-Osler-Weber syndrome) |  | 2 (2) |
| Endocarditis |  | 2 (2) |
| **Management and outcomes** | 0 |  |
| Initial invasive mechanical ventilation |  | 41 (37) |
| ICU mortality |  | 23 (21) |
| In-hospital mortality |  | 27 (25) |
| ICU length of stay, days |  | 7.6 ± 9 |
| In-hospital length of stay, days |  | 26 ± 32 |

Results are expressed as mean ± standard deviation or number of cases (%). Abbreviations: MDCTA, multi-detector computed tomography angiography; ICU, intensive care unit. *Other uncited causes include bronchomediastinal fistula, mucormycosis, and idiopathic arteriovenous malformation (1 case each).

# e-Table 6. Therapeutic procedures performed during hospital stay

| **Variable** | **Missing, n** |  |
| --- | --- | --- |
| **Bronchial arteriography** |  | **725 (77)** |
| Number of procedures |  |  |
| One |  | 595 (63) |
| Two |  | 105 (12) |
| Three |  | 21 (2) |
| Four |  | 4 (0.3) |
| First arteriography results | 8 |  |
| BSH |  | 670 (92) |
| NBSH |  | 90 (12) |
| PA involvement |  | 38 (5) |
| No abnormality |  | 9 (1.2) |
| Technical failure |  | 61/725 (8) |
| Embolization during the first arteriography |  | 690/725 (95) |
| Success |  | 503/690 (73) |
| Recurrence |  | 184/725 (25) |
| Within 24 hours |  | 88/184 (48) |
| Between day 1 to day 7 |  | 77/184 (42) |
| After day 7 |  | 19/184 (10) |
| **Pulmonary angiography** |  | **111 (12)** |
| Number of procedures |  |  |
| One |  | 103 (11) |
| Two |  | 7 (0.7) |
| Three |  | 1 (0.1) |
| First angiography results |  |  |
| Abnormal |  | 95 (86) |
| No abnormality |  | 16 (14) |
| Technical failure |  | 3/111 (3) |
| Vaso occlusion during the first angiography |  | 93/111 (84) |
| Success |  | 74/93 (80) |
| Recurrence |  | 18/111 |
| Within 24 hours |  | 13/18 (54) |
| After 24 hours |  | 5/18 (28) |
| **Surgery*** |  | **60 (10)** |
| Timing |  |  |
| During the ICU stay |  | 45 (75) |
| Within the first 72 hours |  | 17 (28) |
| Type | 1 |  |
| Segmentectomy |  | 2 (3) |
| Lobectomy |  | 36 (60) |
| Pneumonectomy |  | 18 (30) |
| Others |  | 3 (0.5) |
| **Conservative measures**** |  | **618 (65)** |
| Conservative measures only |  | 170 (18) |
| Type |  |  |
| Monitoring only |  | 67 (7) |
| Antibiotics |  | 590 (62) |
| Vasoconstrictors*** |  | 96 (10) |
| Nebulized |  | 37 (4) |
| Intravenous |  | 63 (7) |

Results are expressed as median (IQR) or number of cases (%).

Abbreviations: BSH, bronchial systemic hypervascularization; NBSH, non-bronchial systemic hypervascularization; PA, pulmonary artery; BAE, bronchial arteriography with embolization.

*20 additional patients were operated after hospital discharge.
**Inhaled tranexamic acid was not used in any patient during the study period.
***Both nebulized and intravenous vasoconstrictors, n=4.

# e-Table 7. Adverse events

| **Procedure** |  |
| --- | --- |
| **Bronchial arteriography with embolization** | **115/725 (16)** |
| Major | 47/726 (6) |
| Renal or splenic infarction | 15/726 (2) |
| Stroke | 10/726 (1.4) |
| Acute limb ischemia | 4/726 (0.6) |
| Other | 16/726 (2) |
| Minor | 92/726 (13) |
| Chest, abdominal, facial pain | 37/726 (5) |
| Arterial dissection | 26/726 (4) |
| Puncture site hematoma | 11/726 (2) |
| Other | 20/726 (3) |
| **Surgery** | **25/60 (42)** |
| Haemorrhage | 9/60 (15) |
| ARDS | 3/60 (5) |
| Infection | 15/60 (25) |
| Other | 12/60 (20) |
| **Systemic Vasoconstrictors** | **1/96 (1.1)** |
| Myocardial infarction | 1/96 (1.1) |

Results are expressed as number of cases (%).

Abbreviations: ARDS, acute respiratory distress syndrome.

# e-Table 8. In-hospital mortality by severity score

| **Severity score value** | **0** | **1** | **2** | **3** | **4** | **5** | **6** | **7** |
| --- | --- | --- | --- | --- | --- | --- | --- | --- |
| Patients, n | 308 | 244 | 129 | 149 | 60 | 32 | 18 | 5 |
| Mortality observed, n (%) | 2 (1) | 4 (2) | 8 (6) | 21 (14) | 15 (25) | 18 (56) | 10 (56) | 4 (80) |
| Mortality expected, %* | 1 | 2 | 6 | 16 | 34 | 58 | 79 | 91 |

From Fartoukh *et al.* (5); see e-Table 1.
